# PPy-Coated Mo_3_S_4_/CoMo_2_S_4_ Nanotube-like Heterostructure for High-Performance Lithium Storage

**DOI:** 10.3390/molecules29010234

**Published:** 2023-12-31

**Authors:** Fei Tang, Wei Jiang, Jingjing Xie, Deyang Zhao, Yanfeng Meng, Zhenglong Yang, Zhiqiang Lv, Yanbin Xu, Wenjuan Sun, Ziqiao Jiang

**Affiliations:** School of Chemistry and Materials Science, Ludong University, Yantai 264025, China; tangfeiabca@163.com (F.T.); jweicn@163.com (W.J.); 15684193928@163.com (J.X.); deyang.zhao@ldu.edu.cn (D.Z.); ldumeng@126.com (Y.M.); xuyanbin@ldu.edu.cn (Y.X.); sunwenjuan@ldu.edu.cn (W.S.); jiangziqiao@ldu.edu.cn (Z.J.)

**Keywords:** lithium-ion batteries, heterostructure, nanostructure design, Mo_3_S_4_, CoMo_2_S_4_

## Abstract

Heterostructured materials show great potential to enhance the specific capacity, rate performance and cycling lifespan of lithium-ion batteries owing to their unique interfaces, robust architectures, and synergistic effects. Herein, a polypyrrole (PPy)-coated nanotube-like Mo_3_S_4_/CoMo_2_S_4_ heterostructure is prepared by the hydrothermal and subsequent in situ polymerization methods. The well-designed nanotube-like structure is beneficial to relieve the serious volume changes and facilitate the infiltration of electrolytes during the charge/discharge process. The Mo_3_S_4_/CoMo_2_S_4_ heterostructure could effectively enhance the electrical conductivity and Li^+^ transport kinetics owing to the refined energy band structure and the internal electric field at the heterostructure interface. Moreover, the conductive PPy-coated layer could inhibit the obvious volume expansion like a firm armor and further avoid the pulverization of the active material and aggregation of generated products. Benefiting from the synergistic effects of the well-designed heterostructure and PPy-coated nanotube-like architecture, the prepared Mo_3_S_4_/CoMo_2_S_4_ heterostructure delivers high reversible capacity (1251.3 mAh g^−1^ at 300 mA g^−1^), superior rate performance (340.3 mAh g^−1^ at 5.0 A g^−1^) and excellent cycling lifespan (744.1 mAh g^−1^ after 600 cycles at a current density of 2.0 A g^−1^). Such a design concept provides a promising strategy towards heterostructure materials to enhance their lithium storage performances and boost their practical applications.

## 1. Introduction

The consumption of fossil fuels and the resulting environmental pollution have prompted researchers to explore efficient, clean and sustainable energy storage and conversion technologies. To date, lithium-ion batteries (LIBs), as an outstanding representative of electrochemical energy storage technology, have been widely used in portable electronic devices, hybrid vehicles and large-scale energy storage owing to their high energy/power density and long cycling lifespan [1,2,3,4,5,6]. As a key component of LIBs, anode materials play an important role in their operating voltage, energy density, rate performance, cycling stability, etc. Nevertheless, as the most mature commercial anode material, graphite no longer meets the rapidly growing demand for large capacity and high energy density of LIBs due to its low specific capacity (372 mAh g^−1^) [7,8]. Compared with the graphite anode, transition metal sulfides (TMSs) have received considerable attention due to their high theoretical capacities, suitable operating voltages and excellent electrochemical reversibility [9,10,11,12,13,14]. Generally speaking, TMSs react electrochemically with lithium based on a conversion mechanism to form a metallic substance and Li_2_S, which results in relatively good conductivity and high theoretical capacity [6,15,16,17,18]. Unfortunately, the development of TMSs is restricted by their large volume change during the lithium insertion and extraction. Firstly, the apparent volume change induces serious particle pulverization issues and further loss of effective contact between particles, which in turn leads to rapid capacity decay and poor cycling stability [19,20,21]. Worse still, continuous growth of the solid electrolyte interface caused by particle pulverization issues brings about the electrolyte degradation accompanied with a low Coulombic efficiency. Therefore, it is necessary to enhance the electrochemical performances of TMSs through the elaborate design and modification of the material chemistry and nanostructure.

Compared with the single-component TMSs, the heterostructure consisting of multi-component TMSs [22,23,24,25,26] shows unique advantages: (1) the multi-component heterostructures contribute to a relatively stable product, which is beneficial to relieve structural strain during the charge/discharge process and further enhance their lithium storage performances; (2) the internal electric field at the heterointerfaces is expected to accelerate the charge transfer kinetics, which enables outstanding rate capacities. Recently, various multi-component TMS heterostructures have been constructed to enhance their electrochemical performances. For example, Zheng et al. successfully synthesized CoS_2_-MnS@rGO heterostructures as the anode materials of LIBs [27]. The prepared CoS_2_-MnS@rGO offers a high reversible capacity (1620 mAh g^−1^ at 100 mA g^−1^) and outstanding rate performance (927 mAh g^−1^ at 1.0 A g^−1^). Yan et al. elaborately designed a multilayer FeS_2_@CoS heterostructure encapsulated in a carbon shell (F-FeS_2_@CoS@C) [28]. The F-FeS_2_@CoS@C nanorods deliver an excellent rate capacity (633 mAh g^−1^ at 5.0 A g^−1^) and cycling performance (597.1 mAh g^−1^ at 2.0 A g^−1^ after 800 cycles with the capacity retention ratio of 83.6%).

In addition, nanostructure design has also proved to be an effective strategy for mitigating volume changes of TMSs to achieve a high specific capacity and excellent capacity retention. For example, Xu et al. successfully synthesized FeS_2_/CuS heterostructure nanospheres (HFCSs) [29]. The prepared HFCS electrodes possess a high reversible capacity of 843.3 mAh g^−1^ after 600 cycles at 1.0 A g^−1^ and a surprising rate capability of 530.4 mAh g^−1^ at 10.0 A g^−1^. Liu et al. synthesized a honeycomb-like Co_3_S_4_/MoS_2_ heterostructure with a hollow structure using a hydrothermal method [30]. The reversible capacity of Co_3_S_4_/MoS_2_ is maintained at 365 mAh g^−1^ after 1000 cycles at 1.0 A g^−1^ when used as anode material for LIBs. At even a higher current density of 2.0 A g^−1^, the reversible capacity of 248 mAh g^−1^ can be achieved after 1000 cycles. Zhao et al. successfully synthesized unique CoS-MoS_2_ hollow spheres by using ZIF-67 as the template [31]. The as-prepared composites exhibit good cycling stability with a high reversible specific capacity of 802.4 mAh g^−1^ at a current density of 1.0 A g^−1^ after 400 cycles.

Among TMSs, molybdenum-based sulfides possess excellent electrocatalytic activity, electronic conductivity and abundant redox sites. Therefore, it is feasible to construct Co-Mo-S hybrids as robust anodes for LIBs. However, the nanostructure and chemical composition of Co-Mo-S hybrids still need further modification to improve their electrochemical performances. Moreover, polypyrrole (PPy), known as the conducting polymer, can serve as the coated layers to enhance the structural stability and conductivity of materials. More importantly, the homogeneous PPy-coated layers can also alleviate the giant volume changes and facilitate fast charge transfer during the lithiation-delithiation process. Therefore, it is conceivable that PPy-coated Co-Mo-S heterostructures with a well-designed nanostructure can demonstrate superior electrochemical performance for lithium storage [25,26].

**Scheme 1:**
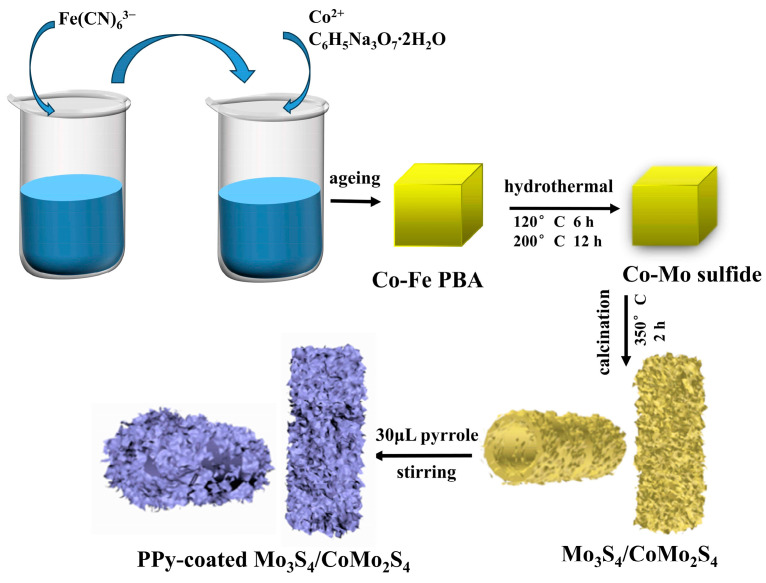
Preparation scheme of PPy-coated Mo_3_S_4_/CoMo_2_S_4_ heterostructure.

Herein, we prepared a PPy-coated Mo_3_S_4_/CoMo_2_S_4_ nanotube-like heterostructure as high-performance anode materials for LIBs by employing Co-Fe Prussian blue as the precursor combined with hydrothermal, calcination and in situ polymerization methods (the specific method is reflected in the Materials and Methods section), as shown in Scheme 1. Based on the morphology, crystal and chemical structure characterization, electrochemical kinetic evaluation and lithium storage performance test, the elaborately designed PPy-coated Mo_3_S_4_/CoMo_2_S_4_ nanotube-like heterostructure presents the following outstanding advantages: (i) the improved electrical conductivity and ion transport kinetics can be realized owing to the refined energy band structure with a smaller band gap and the introduction of the internal electric field at the heterostructure interface; (ii) the strong interactions between Mo_3_S_4_ and CoMo_2_S_4_ are expected to enhance their structural stability; (iii) the nanotube-like structure is conducive to relieving the serious volume changes during the charge/discharge process; (iv) the conductive PPy-coated layer works as armor to inhibit the obvious volume expansion and avoids the pulverization of the active material and aggregation of generated products. Due to the synergetic effect among the well-designed Mo_3_S_4_/CoMo_2_S_4_ heterostructure, nanotube-like structure and conductive PPy-coated layer, the prepared PPy-coated Mo_3_S_4_/CoMo_2_S_4_ heterostructure shows remarkable rate capacity (1251.3 mAh g^−1^ at 300 mA g^−1^; 340.3 mAh g^−1^ at 5.0 A g^−1^) and glorious cycling stability (99.1% capacity retention after 600 cycles at 2.0 A g^−1^), implying its superiority in practical applications.

## 2. Results and Discussion

**Figure 1:**
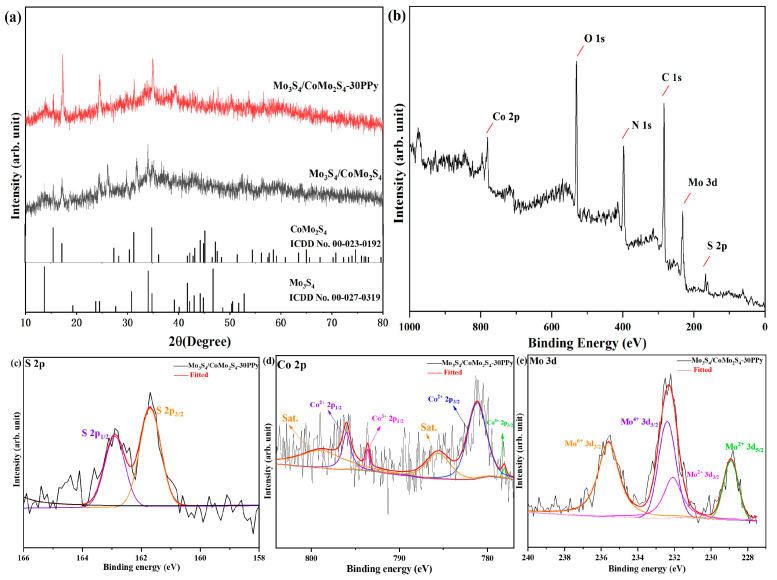
(**a**) XRD patterns of Mo_3_S_4_/CoMo_2_S_4_ and Mo_3_S_4_/CoMo_2_S_4_-30PPy; XPS spectra of Mo_3_S_4_/CoMo_2_S_4_-30PPy: (**b**) survey spectrum; (**c**) S 2p; (**d**) Co 2p; (**e**) Mo 3d.

Figure 1a shows the XRD spectra of Mo_3_S_4_/CoMo_2_S_4_ and Mo_3_S_4_/CoMo_2_S_4_-30PPy. The diffraction peaks at 13.7, 24.5 and 39.1 belong to the (101), (003) and (220) crystal planes of the Mo_3_S_4_ card from the ICDD reference database (PDF No. 00-027-0319), respectively, while the peaks at 15.4, 17.1, 31.2, 34.7 and 42.9 belong to the (002), (101), (−110), (202) and (105) crystal planes of the CoMo_2_S_4_ card from the ICDD reference database (PDF No. 00-023-0192), which suggests the successful construction of the Mo_3_S_4_/CoMo_2_S_4_ heterostructure. To further understand the composition and chemical state of the PPy-coated Mo_3_S_4_/CoMo_2_S_4_ heterostructure, X-ray photoelectron spectroscopy (XPS) was conducted. The XPS spectra reveal the presence of Co (780 eV), Mo (230 eV), S (162 eV), C (285 eV) and N (398 eV) elements in the Mo_3_S_4_/CoMo_2_S_4_-30PPy (Figure 1b). The peak of S 2p is shown in Figure 1c. The main bond of S is the metal–S bond, with the main peak position at 161.8 eV (S 2p_3/2_) and 163.0 eV (S 2p_1/2_) [32]. The high-resolution XPS spectrum of Co 2p (Figure 1d) shows two pairs of spin-orbit split peaks and two satellite peaks. The peaks located at 793.4 and 778.2 eV match the 2p_1/2_ and 2p_3/2_ of Co^3+^, whereas those located at 796.1 and 781.3 eV correspond to 2p_1/2_ and 2p_3/2_ of Co^2+^ [33,34]. For the Mo 3d spectrum (Figure 1e), the peak at 232.4 eV should be assigned to the Mo^4+^ 3d_3/2_, the peaks at 228.9 eV and 232.1 eV should be assigned to the Mo^2+^ 3d_5/2_ and Mo^2+^ 3d_3/2_ peak of Mo in Mo_3_S_4_, and the peak at 235.5 eV should be attributed to Mo^6+^ species in MoO_3_, indicating the surface oxidation of Mo_3_S_4_/CoMo_2_S_4_ [35,36]. TGA measurement was used to investigate the PPy content in the composites (Figure S1). The composite samples were heated from 20 to 800 °C in an air flow environment. The Mo_3_S_4_/CoMo_2_S_4_-30PPy composites display two weight loss regions. The first weight loss in the temperature range of 25~100 °C can be mainly ascribed to the elimination of absorbed H_2_O. The second weight loss appears in the temperature range of 250~700 °C as a strong weight loss, which is related to the combustion reaction of PPy chains. According to the TGA analysis, it is indicated that the PPy content in the Mo_3_S_4_/CoMo_2_S_4_-30PPy is 40%.

**Figure 2:**
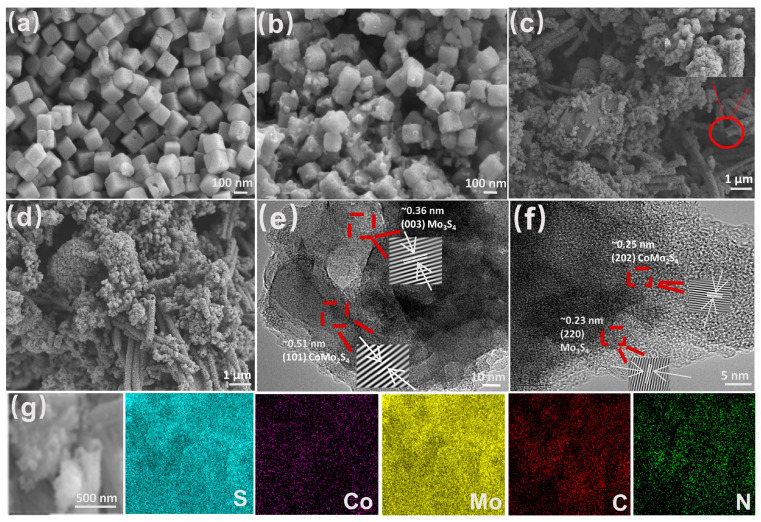
SEM images of samples: (**a**) Co-Fe PBA, (**b**) Co-Mo sulfide intermediate, (**c**) Mo_3_S_4/_CoMo_2_S_4_, (**d**) Mo_3_S_4_/CoMo_2_S_4_-30PPy; (**e**,**f**) HRTEM images and (**g**) element mapping of Mo_3_S_4_/CoMo_2_S_4_-30PPy.

The morphology and microstructure of the prepared Co-Fe PBA precursor, Co-Mo sulfide intermediate and Mo_3_S_4_/CoMo_2_S_4_-30PPy were investigated using SEM. As shown in Figure 2a, the Co-Fe PBA precursor presents uniform cubes with a relatively smooth surface and sharp edges and with an average size of ~120 nm. As shown in Figure 2b, the Co-Mo sulfide intermediate with similar cubes (~120 nm) was formed through an ionic exchange reaction between Fe(CN)_6_^3−^ in Co-Fe PBA and S^2−^ during the hydrothermal process [37]. To obtain the Mo_3_S_4_/CoMo_2_S_4_ heterostructure, high-temperature calcination was conducted. During the calcination, two different morphologies of particles are formed: some are hollow nanotubes, and others are cubic particles (Figure 2c). The formation of hollow tubular structures (diameter of ~150 nm) is attributed to solid-state reactions caused by the interdiffusion of metal sulfides at high temperatures (the regular cubes of the Co-Mo sulfide intermediate are decomposed, with the generation of hollow nanotubes), which is beneficial to relieve the serious volume changes of Mo_3_S_4_/CoMo_2_S_4_ during the charge/discharge process and further boost the excellent cycling stability; the other part remains in the cubic structure of the precursor and is brought together through a series of reactions. In order to enhance the electrical conductivity of the Mo_3_S_4_/CoMo_2_S_4_ heterostructure, the highly conductive PPy layer was coated on the nanotube-like Mo_3_S_4_/CoMo_2_S_4_ heterostructure (diameter of ~300 nm). As shown in Figure 2d, compared with the Mo_3_S_4_/CoMo_2_S_4_ heterostructure, a relatively rough surfaces and porous structure can be found for Mo_3_S_4_/CoMo_2_S_4_-30PPy. The rough surfaces and porous structure are expected to enhance electrolyte penetration, which could contribute to efficient ion diffusion kinetics. Moreover, PPy could work as armor to inhibit the obvious volume expansion and avoids the pulverization of the active material and aggregation of generated products, which is conducive to enhancing the cycling stability of the Mo_3_S_4_/CoMo_2_S_4_ heterostructure. HRTEM images of Mo_3_S_4_/CoMo_2_S_4_-30PPy are shown in Figure 2e,f. The lattice spacing of 0.36 and 0.23 nm can be attributed to the (003) and (220) crystal planes of Mo_3_S_4_, while the lattice spacing of 0.51 and 0.25 nm corresponds to the (101) and (202) crystal planes of CoMo_2_S_4_, which further confirms the successful formation of the Mo_3_S_4_/CoMo_2_S_4_ heterostructure. In addition, a relatively complete conductive network was coated on the surface of the Mo_3_S_4_/CoMo_2_S_4_ heterostructure after the in situ polymerization of pyrrole, which further improves the electrical conductivity of the Mo_3_S_4_/CoMo_2_S_4_ heterostructure. The element mapping (Figure 2g) shows the presence and uniform distribution of Mo, S, C, Co and N elements in the PPy-coated Mo_3_S_4_/CoMo_2_S_4_ heterostructure. The EDX spectrum of Mo_3_S_4_/CoMo_2_S_4_ and Mo_3_S_4_/CoMo_2_S_4_-30PPy (Figure S2) shows that the atomic ratio of Co: Mo: S is about 1: 5: 8. Combined with XRD and HRTEM images, the Mo_3_S_4_/CoMo_2_S_4_ nanotube-like heterostructure was successfully synthesized.

**Figure 3:**
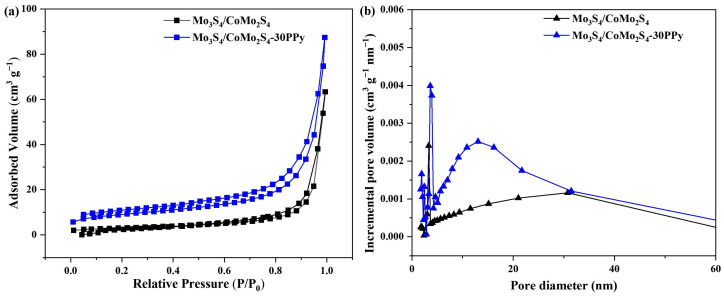
(**a**) N_2_ adsorption–desorption isotherm and (**b**) aperture distribution of Mo_3_S_4_/CoMo_2_S_4_ and Mo_3_S_4_/CoMo_2_S_4_-30PPy samples.

The specific area and pore size distribution of the synthesized samples were investigated by nitrogen adsorption–desorption isotherms. As shown in Figure 3a, the nitrogen adsorption–desorption isotherms reveal type IV isotherms due to the hysteresis lines in the range of 0.5–1.0 P/P_0_, which suggests there are abundant mesopores in the prepared samples. Specifically, the measured BET specific surface areas of Mo_3_S_4_/CoMo_2_S_4_ and Mo_3_S_4_/CoMo_2_S_4_-30PPy are 10.55 and 32.10 m^2^ g^−1^, and the pore volumes are 0.034 and 0.068 cm^3^ g^−1^, respectively. The pore size distribution curves are shown in Figure 3b. It can be seen that both samples have mesoporous structures, with a size distribution of 2–7 nm. High specific surface areas and plentiful mesopores ensure easy electrolyte penetration and ion transport, which is beneficial to enhance their rate capability.

**Figure 4:**
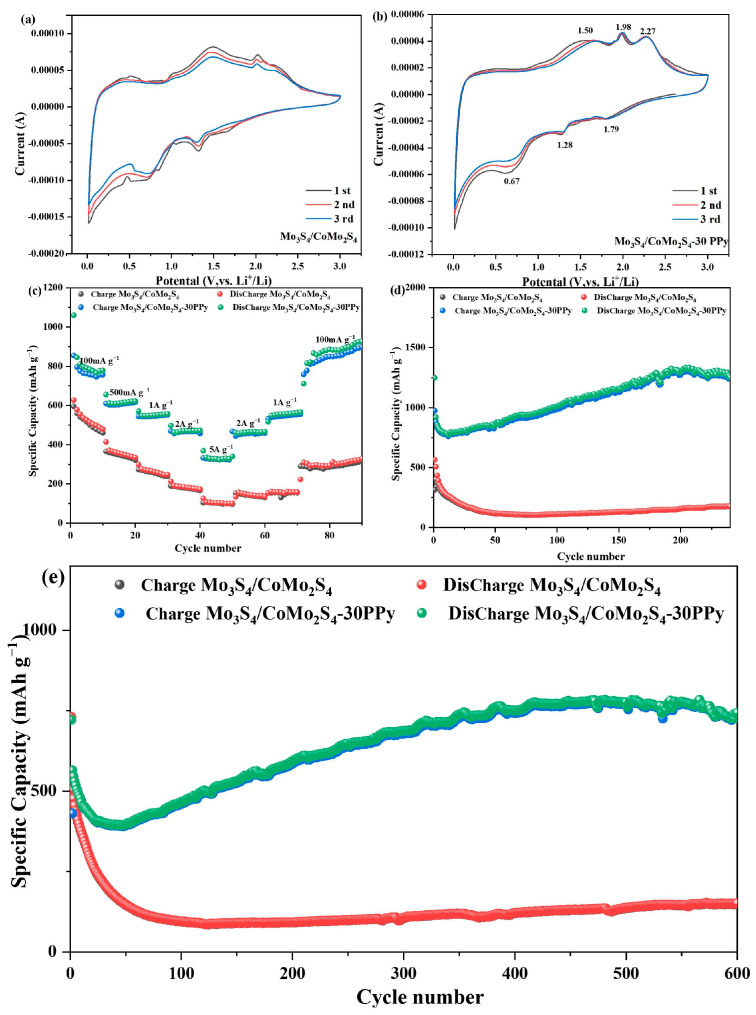
(**a**,**b**) CV curves at 0.1 mV s^−1^; (**c**) rate performances at different current densities; (**d**,**e**) cycling performances at 300 mA g^−1^ and 2.0 A g^−1^ of Mo_3_S_4_/CoMo_2_S_4_ and Mo_3_S_4_/CoMo_2_S_4_-30PPy.

In order to evaluate the lithium storage performances of the prepared samples, cyclic voltammetry (CV) was measured at 0.1 mV s^−1^, with a potential window of 0.01–3.00 V (vs. Li/Li^+^). As shown in Figure 4b, an obvious peak at 1.28 V can be observed in the first cathodic scan, which is ascribed to the insertion of Li^+^ into the active material lattice, accompanying the formation of Li_x_MoS and Li_y_CoMo_2_S_4_ (xLi^+^ + xe^−^ + MoS → Li_x_MoS; yLi^+^ + ye^−^ + CoMo_2_S_4_ → Li_y_CoMo_2_S_4_) [38]. In addition, the peak at 0.67 V is indexed to the further reduction of Li_x_MoS and Li_y_CoMo_2_S_4_ (Li_x_MoS → Li_x_S + Mo; Li_y_CoMo_2_S_4_ → 4Li_0.25y_Co + 2Mo) and the formation of a solid electrolyte interface (SEI) [39]. The peak at 1.79 V results from the lithium insertion into different defect sites of the active materials [40]. The anodic peak at 1.50 V is likely due to the de-lithiation of residual Li_x_MoS and Li_y_CoMo_2_S_4_ [41]. The oxidation peaks at 1.98 and 2.27 V can be attributed to the oxidation of Li_2_S (nLi_2_S ↔ S_n_^2-^+ 2nLi^+^ + (2n − 2)e^−^) [42]. Notably, the overlap of the CV curves in the first three cycles indicates the good reversibility and stability of the Mo_3_S_4_/CoMo_2_S_4_-30PPy electrodes. Figure S3 represents the galvanostatic charge and discharge curve of Mo_3_S_4_/CoMo_2_S_4_ and Mo_3_S_4_/CoMo_2_S_4_-30PPy at a current density of 300 mA g^−1^. The initial discharging-voltage curve of Mo_3_S_4_/CoMo_2_S_4_-30PPy electrodes is similar to that of Mo_3_S_4_/CoMo_2_S_4_. It shows a voltage plateau at approximately 1.3 V and 0.7 V. The plateau at 1.3 V may be attributed to the electrochemical conversion reaction of Li^+^ into the active material lattice and the formation of Li_x_MoS and Li_y_CoMo_2_S_4_, while the one at 0.7 V should be ascribed to the formation of SEI film and the further reduction of Li_x_MoS and Li_y_CoMo_2_S_4_. This is consistent with the CV test results above. In addition, the charging curves for three cycles almost overlapped, which indicates the excellent cycle stability of the electrode.

Figure 4c shows the rate performances of the two electrodes at different current densities. The Mo_3_S_4_/CoMo_2_S_4_ electrode presents a discharge capacity of 515.3 mAh g^−1^ at 100 mA g^−1^. With increasing of the current density from 100 mA g^−1^ to 5.0 A g^−1^, a dramatic decreasing discharge capacity of 515.3, 353.1, 261.7, 182.9 and 104.2 mAh g^−1^ is observed. In contrast, the Mo_3_S_4_/CoMo_2_S_4_-30PPy electrode had excellent rate capacities of 785.0, 622.7, 527.2, 472.8 and 340.3 mAh g^−1^ with the increase in current densities from 100 mA g^−1^ to 5.0 A g^−1^. When the current density recovers to 100 mA g^−1^, a high reversible capacity of 926.4 mAh g^−1^ can be achieved, suggesting a glorious capacity recovery ability. The improved rate capacities can be ascribed to the PPy-coated nanotube-like heterostructure, which contributes to the enhanced electrical conductivity and ion transport kinetics. Figure 4d,e shows the cycling performance of the two electrodes. It can be seen that the Mo_3_S_4_/CoMo_2_S_4_-30PPy electrode shows an impressive reversible capacity of 1251 mAh g^−1^ at 300 mA g^−1^ after 240 cycles and presents almost 100% capacity retention, while the discharge capacity of the Mo_3_S_4_/CoMo_2_S_4_ electrode is only 285.7 mAh g^−1^ at 300 mA g^−1^ after 240 cycles. To fully investigate their cycling performances, the long cycling performances at 2 A g^−1^ were also investigated. Mo_3_S_4_/CoMo_2_S_4_-30PPy (Figure 4e) can maintain a stable discharge capacity of 744.1 mAh g^−1^ after 600 cycles, while the specific capacity of the Mo_3_S_4_/CoMo_2_S_4_ electrode is only 151.6 mAh g^−1^. Figure S4 shows the SEM images of the Mo_3_S_4_/CoMo_2_S_4_ and Mo_3_S_4_/CoMo_2_S_4_-30PPy electrode after 10 discharge/charge cycles at 300 mA g^−1^. Compared with the original morphology, the material retains its nanotube structure after cycling. This result can positively corroborate the effective role of the atomic heterointerface and PPy as a protecting layer to alleviate the structural collapse. We compared previous similar experiments in Table S1 [39,43,44,45,46,47]. It was found that the rate capacities and cyclic stability of Mo_3_S_4_/CoMo_2_S_4_-30PPy are improved, indicating the superiority of our designs. The impressive retention capability of Mo_3_S_4_/CoMo_2_S_4_-30PPy can be ascribed to the PPy-coated nanotube-like heterostructure. On the one hand, the hollow nanotube-like structure is beneficial for addressing the serious volume changes during the charge/discharge process; on the other hand, the conductive PPy-coated layer can inhibit the obvious volume expansion and avoid the pulverization of the active material and aggregation of the generated products. We should point out that the specific capacity of Mo_3_S_4_/CoMo_2_S_4_-30PPy is higher than the theoretical values of Mo_3_S_4_ and CoMo_2_S_4_ in LIBs. This phenomenon often happens for TMSs, which may be attributed to the repeated generation of the polymeric membrane and possibly interfacial lithium storage as well as electrochemical activation of the hybrid during the cycling process [45,48,49].

**Figure 5:**
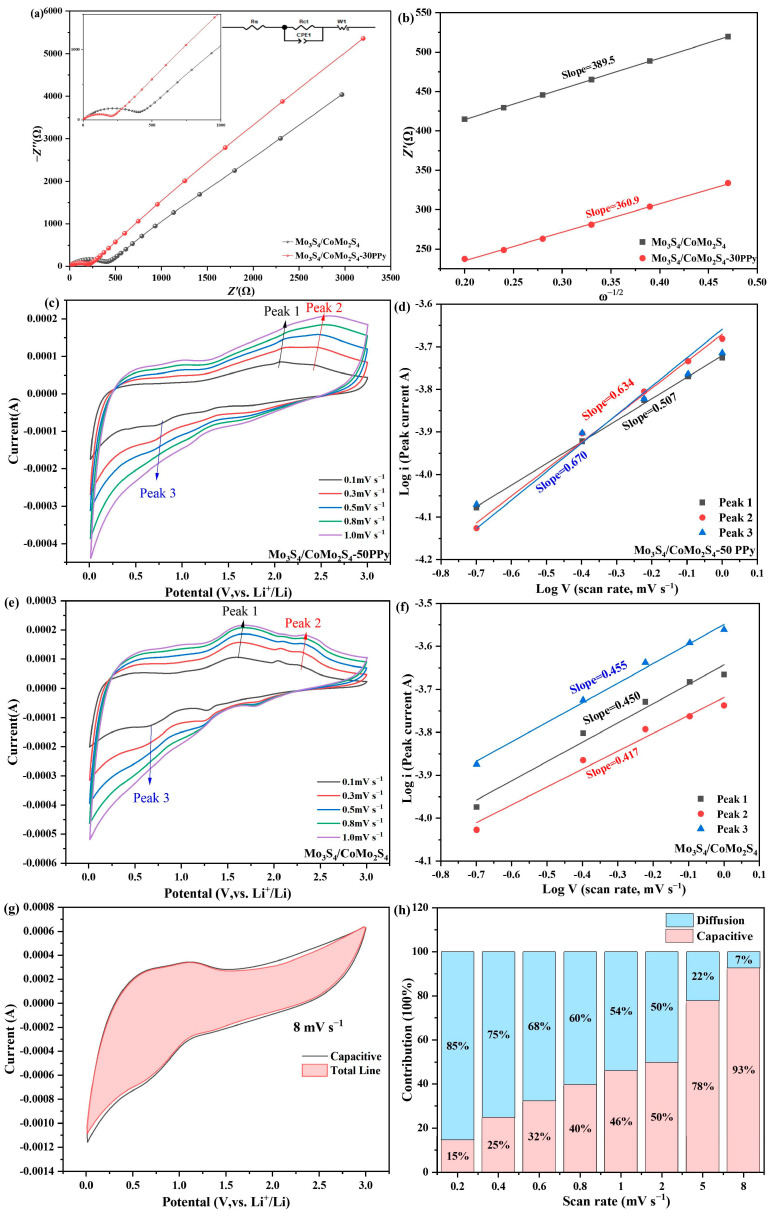
(**a**,**b**) Nyquist plots and the relationships between Z’ and ω^−1/2^ in low-frequency region of Mo_3_S_4_/CoMo_2_S_4_, Mo_3_S_4_/CoMo_2_S_4_-30PPy; (**c**,**e**) CV curves at scan rates from 0.2 to 1.0 mV s^−1^ and (**d**,**f**) the relationships between log *v* and log *i* plots for cathodic and anodic peaks of Mo_3_S_4_/CoMo_2_S_4_-30PPy and Mo_3_S_4_/CoMo_2_S_4_; (**g**) ratio of the capacitive-controlled charge contribution (shaded area) to the total current at a scan rate of 8 mV s^−1^; and (**h**) pseudo-capacitance contribution under different scan rates of Mo_3_S_4_/CoMo_2_S_4_-30PPy.

To investigate the reason for the outstanding rate capacities of the Mo_3_S_4_/CoMo_2_S_4_-30PPy electrode, electrochemical impedance spectra (EIS) measurements were conducted (Figure 5a,b). The Nyquist plots consist of a semicircle in the high-frequency region and a slant line in the low-frequency region, which correspond to the charge transfer resistance between the electrolyte and electrodes, *R*_ct_, and the Warburg resistance. The former is usually used to detect the electrochemical reaction kinetics, while the latter is closely related to the Li^+^ diffusion inside the active materials. The Li^+^ diffusion coefficient of the Mo_3_S_4_/CoMo_2_S_4_-30PPy electrode reaches up to 3.05 × 10^−11^ cm^2^ s^−1^ according to EISs, much higher than that of the Mo_3_S_4_/CoMo_2_S_4_ electrode (2.62 × 10^−11^ cm^2^ s^−1^), which implies much faster ion diffusion of the Mo_3_S_4_/CoMo_2_S_4_-30PPy electrode owing to the unique PPy-coated nanotube-like heterostructure. As a consequence, the charge transfer kinetics are dramatically enhanced for the Mo_3_S_4_/CoMo_2_S_4_-30PPy electrode owing to its much lower *R*_ct_ (221.3 Ω vs. 464.8 Ω), indicating accelerated charge transport behaviors and further boosted electrochemical reactions.

To gain further insight into the lithium storage behaviors of the prepared electrodes, CV tests at different sweep speeds (0.2 mV s^−1^; 0.4 mV s^−1^; 0.6 mV s^−1^; 0.8 mV s^−1^; 1 mV s^−1^) were conducted. With the gradient increase of the sweep speeds, all of the CV curves present similar shapes (Figure 5c,e), indicating good electrochemical reversibility of the prepared electrodes.

In general, the relationship between current (*i*) and sweep speed (*v*) is in accordance with Equation (1):

(1) $$i=av^{b}$$

where *a* and *b* are the adjustable parameters. Generally, a value of *b* close to 0.5 indicates a diffusion-controlled process, while a value of *b* close to 1 indicates a pseudo-capacitance-controlled process [50]. For the Mo_3_S_4_/CoMo_2_S_4_-30PPy electrode (Figure 5c,d), the b values of the cathodic and anodic peaks are 0.507, 0.632 and 0.670, indicating a combination of diffusion-controlled and pseudocapacitance-controlled processes. Whereas for the Mo_3_S_4_/CoMo_2_S_4_ electrode (Figure 5e,f), the *b*-values of the cathodic and anodic peaks are 0.455, 0.450 and 0.417, which are much smaller than those of the Mo_3_S_4_/CoMo_2_S_4_-30PPy electrode, indicating a complete diffusion-controlled process. The specific capacitive contribution for the Mo_3_S_4_/CoMo_2_S_4_-30PPy electrode was investigated and is shown in Figure 5g,h. With increasing of the scan rate, the capacitive contribution increases. The percentages of capacitive contributions are 15%, 25%, 32%, 40%, 46%, 50%, 78% and 93% at 0.2, 0.4, 0.6, 0.8, 1, 2, 5 and 8 mV s^−1^, respectively. Therefore, it can be concluded that the PPy-coated nanotube-like heterostructure can greatly improve the pseudo-capacitive behavior and accelerate the electrochemical reaction kinetics.

## 3. Materials and Methods

### 3.1. Materials

All the chemicals used were analytical grade and were used without further purification: Sodium 4-methylbenzenesulfonate (GUANGFU, Tianjin, China, 98%), Cobalt hexahydrate chloride (VETEC, Speyer, Germany, 99%), Trisodium citrate (FUNCHEN, Tianjin, China, 99%), Potassium ferricyanide (BEILIANJINGXI, Tianjin, China, 99.5%), Sodium molybdate dihydrate (Boddi, Tianjin, China, 99%), Thiourea (ALADDIN, Shanghai, China, 99%), Pyrrole (MACKLIN, Shanghai, China, 99%), Ethanol absolute (MACKLIN, Shanghai, China, 95%).

### 3.2. Preparation of Co-Fe Prussian Blue (Co-Fe PBA)

A total of 0.5 mmol of K_3_[Fe (CN)_6_] was dissolved in 20 mL of deionized water (solution A). A total of 0.5 mmol of CoCl_2_∙6H_2_O and 1 mmol of sodium citrate dihydrate were dissolved in 20 mL of deionized water (solution B). Then, solutions A and B were mixed and stirred for 24 h to form a precipitate. After aging for 12 h, the precipitate was centrifuged, washed and dried.

### 3.3. Preparation of Mo_3_S_4_/CoMo_2_S_4_ Heterostructure

A total of 1 mmol of Co-Fe PBA was dispersed into 50 mL of ethanol and sonicated for 30 min. Then, 15 mL of ethanol containing 3 mmol of thiourea was added to the above solution and stirred for 30 min. Subsequently, 15 mL of deionized water containing 1 mmol of Na_2_MoO_4_∙2H_2_O was added and stirred for an additional 10 min. The mixed solution was sealed in a 100 mL autoclave and kept at 120 °C for 6 h and then 200 °C for 12 h. After being washed and dried, the collected product was calcined at 350 °C for 2 h, with a heating rate of 2 °C/min, under an Ar atmosphere to obtain Mo_3_S_4_/CoMo_2_S_4_ composites.

### 3.4. Preparation of PPy-Coated Mo_3_S_4_/CoMo_2_S_4_ Heterostructure

A total of 0.05 g of sodium 4-methylbenzenesulfonate was dispersed in 40 mL of deionized water. Then, 30 μL of pyrrole monomer was moved into the solution and sonicated for 10 min. Thereafter, 0.1 g of Mo_3_S_4_/CoMo_2_S_4_ powder was added to the above dispersions and stirred for 2 h. Next, 10 mL of deionized water containing 0.12 g of FeCl_3_ was slowly added and stirred for 4 h. Finally, the resulting mixture was centrifuged, washed and dried. The resulting Mo_3_S_4_/CoMo_2_S_4_-PPy composites were denoted as Mo_3_S_4_/CoMo_2_S_4_-30PPy.

### 3.5. Characterization

The composition and crystal structure of the products were analyzed by X-ray diffraction (XRD, D8 Advance, Karlsruhe, Germany), with Cu-Kα as the radiation source. The morphology and microstructure were analyzed by scanning electron microscopy (SEM, Hitachi S4880, Tokyo, Japan) and transmission electron microscopy (TEM, Tecnai G2 F30, Hillsboro, OR, USA). X-ray photoelectron spectroscopy (XPS, Thermo ESCALAB 250XI, Waltham, MA, USA) was used to analyze their chemical compositions, using Al-Kα as the radiation source. The specific surface area and pore size distribution were obtained by nitrogen adsorption–desorption analysis (ASAP 2460, Shanghai, China).

### 3.6. Electrochemical Measurement

The prepared samples were mixed with Super P and polyvinylidene fluoride (PVDF) and dispersed in N-methyl-2-pyrrolidone (NMP), with a mass ratio of 70:15:15. The slurry was coated on the copper foil, with a mass loading of 0.6 mg cm^−2^, and then dried at 80 °C overnight. The coin cells were assembled in the glove box, with lithium foil (0.6 mm) as the counter electrode, Celgard 2400 as the separator and 40 µL of 1.0 mol L^−1^ LiPF_6_ (EC:DMC:EMC = 1:1:1) as the electrolyte. The Neware CT-3008 battery test system was used for the galvanostatic charge and discharge analysis, with a voltage window of 0.01 to 3.0 V. Cyclic voltammetry (CV) at a scan rate of 0.1 mV s^−1^ and electrochemical impedance spectroscopy (EIS) with the frequency from 0.01 Hz to 100 kHz were conducted using the Zahner Pro electrochemical workstation.

## 4. Conclusions

In conclusion, we successfully designed and prepared a PPy-coated Mo_3_S_4_/CoMo_2_S_4_ nanotube-like heterostructure using the hydrothermal, calcination and subsequent polymerization methods. On the one hand, the Mo_3_S_4_/CoMo_2_S_4_ heterostructure could effectively enhance the electrical conductivity and Li^+^ transport kinetics owing to the refined energy band structure and the internal electric field at the heterostructure interface; on the other hand, the well-designed nanotube-like structure is beneficial for relieving the serious volume changes and facilitating the infiltration of electrolytes during the charge/discharge process. Moreover, the conductive PPy-coated layer could inhibit the obvious volume expansion like a firm armor and further avoid the pulverization of the active material and the aggregation of generated products. As a consequence, the elaborately prepared Mo_3_S_4_/CoMo_2_S_4_-30PPy nanotube-like heterostructure delivers a high reversible capacity of 1251.3 mAh g^−1^ at 300 mA g^−1^ and impressive rate capacity (340.3 mAh g^−1^ at 5.0 A g^−1^). In addition, an excellent cycling lifespan (744.1 mAh g^−1^ after 600 cycles at a current density of 2.0 A g^−1^) can be also achieved. These results clearly exhibit a promising strategy towards the rational design of heterostructure materials to enhance the lithium storage performance of transition metal sulfides and boost their practical applications.

## Data Availability

The data presented in this study are available in the Supplementary Materials.

## Supplementary Materials

The following supporting information can be downloaded at: https://www.mdpi.com/article/10.3390/molecules29010234/s1. Figure S1: TGA curves of Mo_3_S_4_/CoMo_2_S_4_-30PPy. Figure S2: EDX image of Mo_3_S_4_/CoMo_2_S_4_-30PPy. Figure S3: Galvanostatic charge/discharge voltage profiles of (a) Mo_3_S_4_/CoMo_2_S_4_, (b) Mo_3_S_4_/CoMo_2_S_4_-30PPy. Figure S4 SEM images of (a) Mo_3_S_4_/CoMo_2_S_4_, (b) Mo_3_S_4_/CoMo_2_S_4_-30PPy electrode as anode of LIBs after 10 cycles at 300 mA g^−1^. Table S1: Electrochemical performance of the Li-ion batteries with similar studies in previous reported literatures.
