# Supplementary material for: PPy-Coated Mo3S4/CoMo2S4 Nanotube-like Heterostructure for High-Performance Lithium Storage
Source: Molecules. 2023 Dec 31;29(1):234. doi: 10.3390/molecules29010234 (PMC10780578; doi:10.3390/molecules29010234)
Supplement: Supplementary file 1 [file molecules-29-00234-s001.zip › molecules-2740030-supplementary.pdf]

# Supporting Information

# PPy Coated $Mo_3S_4$/$CoMo_2S_4$ Nanotube-Like Heterostructure for High-Performance Lithium Storage

**Fei Tang [1], Wei Jiang [1], Jingjing Xie [1], Deyang Zhao [1], Yanfeng Meng [1], Zhenglong Yang [1,*], Zhiqiang Lv [1,*], Yanbin Xu [1,*], Wenjuan Sun [1] and Ziqiao Jiang [1]**

[1] School of Chemistry and Materials Science, Ludong University, Yantai 264025, China

* Correspondence: yzl@iccas.ac.cn (Z.Y.); lzq268197@163.com (Z.L.); xuyanbin@ldu.edu.cn (Y.X.)

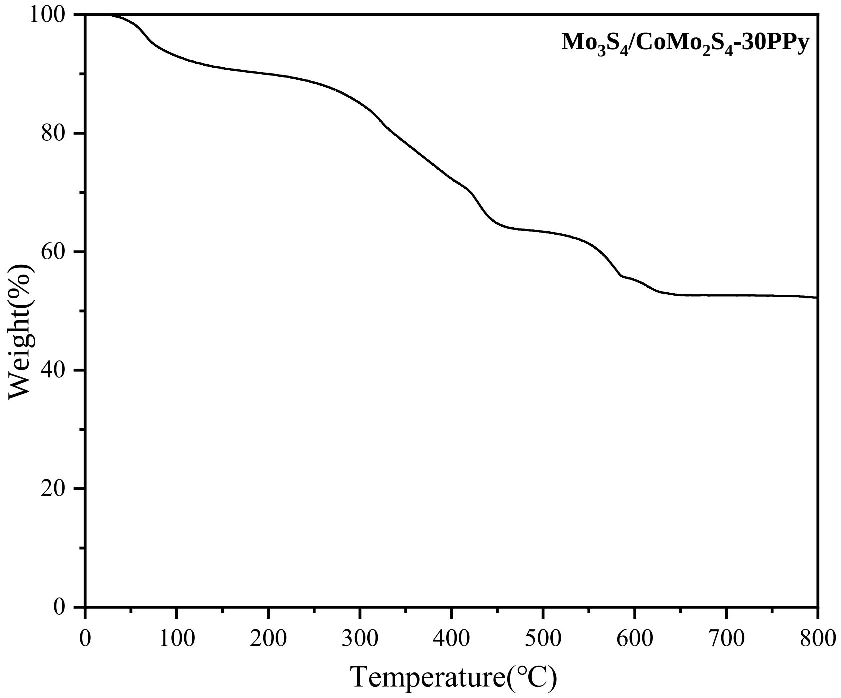


**Figure S1** TGA curves of $Mo_3S_4/CoMo_2S_4$-30PPy.

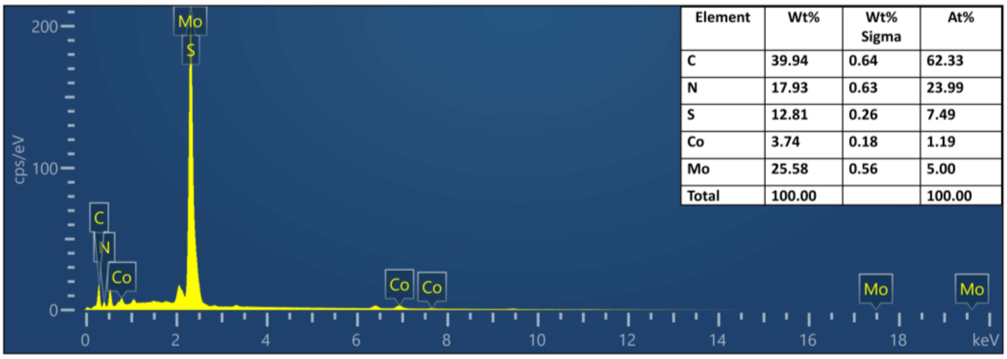


| Element | Wt% | Wt% Sigma | At% |
|---|---|---|---|
| C | 39.94 | 0.64 | 62.33 |
| N | 17.93 | 0.63 | 23.99 |
| S | 12.81 | 0.26 | 7.49 |
| Co | 3.74 | 0.18 | 1.19 |
| Mo | 25.58 | 0.56 | 5.00 |
| Total | 100.00 | | 100.00 |

**Figure S2** EDX image of $Mo_3S_4/CoMo_2S_4$-30PPy.

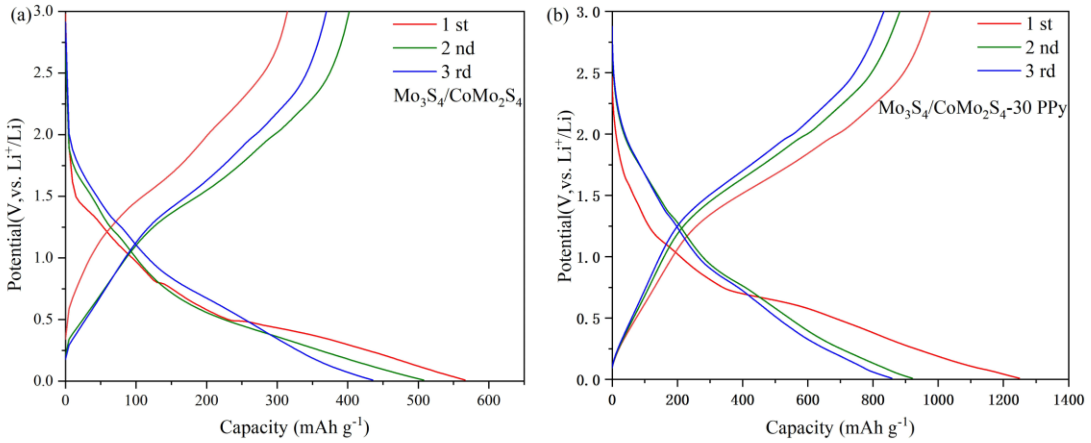


**Figure S3** Galvanostatic charge/discharge voltage profiles of (a)$Mo_3S_4/CoMo_2S_4$, (b)$Mo_3S_4/CoMo_2S_4$-30PPy.

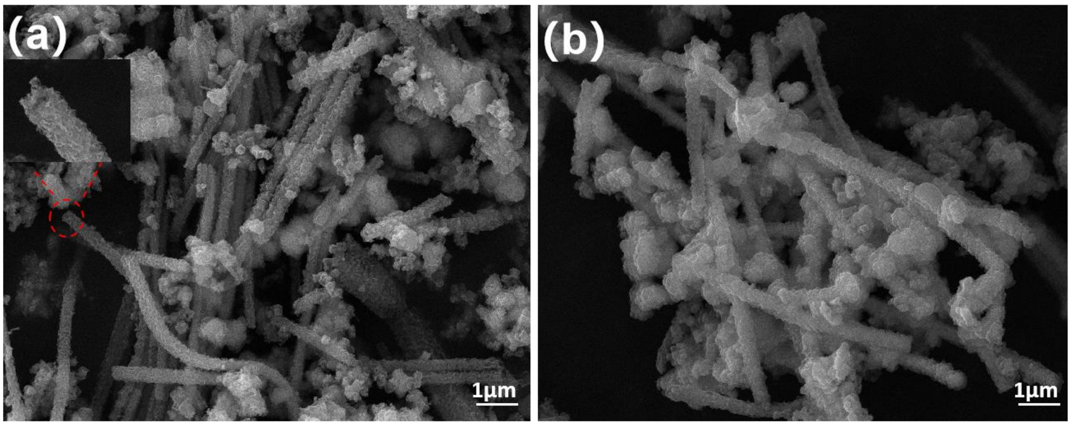


**Figure S4** SEM images of (a)$Mo_3S_4/CoMo_2S_4$, (b)$Mo_3S_4/CoMo_2S_4$-30PPy electrode as anode of LIBs after 10 cycles at 300 mA $g^{-1}$.

**Table S1** Electrochemical performance of the Li-ion batteries with similar studies in previous reported literatures.

| Materials | Initial discharge specific capacity | Rate performance | Cycling stablity | Ref. |
|---|---|---|---|---|
| **CoMoS@C** | 1164 mAh $g^{-1}$ at 0.5 A $g^{-1}$ | 425 mAh $g^{-1}$ at 1A $g^{-1}$ | 715 mAh $g^{-1}$ after 200 cycles at 0.5 A $g^{-1}$ | [43] |
| **$Co_3S_4$/$CoMo_2S_4$@rGO** | 823.0 mAh $g^{-1}$ at 0.2A $g^{-1}$ | 317.2mAh $g^{-1}$ at 1.0A $g^{-1}$ | 595.4 mAh $g^{-1}$ after 100 cycles at 0.2A $g^{-1}$ | [44] |
| **C@$MoS_2$@PPy** | 1050 mAh $g^{-1}$ at 200 μA $cm^{-2}$ | 630 mA h $g^{-1}$ at 1000 μA $cm^{-2}$ | 805 mAh $g^{-1}$ after 200 cycles at 200 μA $cm^{-2}$ | [45] |
| **$MoS_2$/$CoMo_2S_4$/$Co_3S_4$** | 1345 mAh $g^{-1}$ at 0.2A $g^{-1}$ | 360mAh $g^{-1}$at 10A $g^{-1}$ | 770 mAh $g^{-1}$ after 200 cycles at 0.2A $g^{-1}$ | [39] |
| **$MoS_2$-PPY-rGO** | 1428 mAh $g^{-1}$ at 0.2A $g^{-1}$ | 600mAh $g^{-1}$ at 2.0A $g^{-1}$ | 1070 mAh $g^{-1}$ after 400 cycles at 0.2A $g^{-1}$ | [46] |
| **$MoS_2$/SnS/CoS** | 875.0 mAh $g^{-1}$ at 0.1A $g^{-1}$ | 304.9 mAh $g^{-1}$ at 5 A $g^{-1}$ | 627.6 mA h $g^{-1}$ after 100 cycles at 0.1 A $g^{-1}$ | [47] |
| **$Mo_3S_4$/$CoMo_2S_4$-30PPy** | 1348.8 mAh $g^{-1}$ at 0.3 A $g^{-1}$ | 744. mAh $g^{-1}$ at 2A $g^{-1}$ | 1251 mA h $g^{-1}$ after 240 cycles at 0.3 A $g^{-1}$ | This work |


39. Wang, P.; Zhang, P.; Zheng, X.; Cao, J.; Liu, Y.; Feng, J.; Qi, J. Constructing $MoS_2$/$CoMo_2S_4$/$Co_3S_4$ nanostructures supported by graphene layers as the anode for lithium-ion batteries. *Dalton Trans.* **2020,** 49, 1167-1172. DOI: 10.1039/c9dt04042k.

43. Dominguez, N.; Torres, B.; Barrera, L. A.; Rincon, J. E.; Lin, Y.; Chianelli, R. R.; Ahsan, M. A.; Noveron, J. C. Bimetallic CoMoS Composite Anchored to Biocarbon Fibers as a High-Capacity Anode for Li-Ion Batteries. *ACS Omega* **2018,** 3, 10243-10249. DOI: 10.1021/acsomega.8b00654.

44. Liao, Y.; Wu, C.; Zhong, Y.; Chen, M.; Cai, L.; Wang, H.; Liu, X.; Cao, G.; Li, W. Highly dispersed Co-Mo sulfide nanoparticles on reduced graphene oxide for lithium and sodium ion storage. *Nano Res.* **2020,** 13, 188-195. DOI: 10.1007/s12274-019-2594-2.

45. Lu, B.; Liu, J.; Hu, R.; Wang, H.; Liu, J.; Zhu, M. C@$MoS_2$@PPy sandwich-like nanotube arrays as an ultrastable and high-rate flexible anode for Li/Na-ion batteries. *Energy Storage Materials* **2018,** 14, 118-128. DOI: 10.1016/j.ensm.2018.02.022.

46. Xie, D.; Wang, D. H.; Tang, W. J.; Xia, X. H.; Zhang, Y. J.; Wang, X. L.; Gu, C. D.; Tu, J. P. Binder-free network-enabled $MoS_2$-PPY-rGO ternary electrode for high capacity and excellent stability of lithium storage. *Journal of Power Sources* **2016,** 307, 510-518. DOI: 10.1016/j.jpowsour.2016.01.024.

47. Zhang, R.; Dong, Y.; Su, Y.; Zhai, W.; Xu, S. $MoS_2$/SnS/CoS Heterostructures on Graphene: Lattice-Confinement Synthesis and Boosted Sodium Storage. *Molecules* **2023,** 28. DOI: 10.3390/molecules28165972.
